# Dorsal root ganglia in Friedreich ataxia: satellite cell proliferation and inflammation

**DOI:** 10.1186/s40478-016-0288-5

**Published:** 2016-05-03

**Authors:** Arnulf H. Koeppen, R. Liane Ramirez, Alyssa B. Becker, Joseph E. Mazurkiewicz

**Affiliations:** Research Service, Veterans Affairs Medical Center, Albany, NY USA; Departments of Neurology and Pathology, Albany Medical College, Albany, NY USA; Center for Neuropharmacology and Neuroscience, Albany Medical College, Albany, NY USA

**Keywords:** Dorsal root ganglion, Friedreich ataxia, Gap junction, Inflammation, Monocyte, Satellite cell

## Abstract

**Introduction:**

Dorsal root ganglia (DRG) are highly vulnerable to frataxin deficiency in Friedreich ataxia (FA), an autosomal recessive disease due to pathogenic homozygous guanine-adenine-adenine trinucleotide repeat expansions in intron 1 of the *FXN* gene (chromosome 9q21.11). An immunohistochemical and immunofluorescence study of DRG in 15 FA cases and 12 controls revealed that FA causes major primary changes in satellite cells and inflammatory destruction of neurons. A panel of antibodies was used to reveal the cytoplasm of satellite cells (glutamine synthetase, S100, metabotropic glutamate receptors 2/3, excitatory amino acid transporter 1, ATP-sensitive inward rectifier potassium channel 10, and cytosolic ferritin), gap junctions (connexin 43), basement membranes (laminin), mitochondria (ATP synthase subunit beta and frataxin), and monocytes (CD68 and IBA1).

**Results:**

Reaction product of the cytoplasmic markers and laminin confirmed proliferation of satellite cells and processes into multiple perineuronal layers and residual nodules. The formation of connexin 43-reactive gap junctions between satellite cells was strongly upregulated. Proliferating satellite cells in FA displayed many more frataxin- and ATP5B-reactive mitochondria than normal. Monocytes entered into the satellite cell layer, appeared to penetrate neuronal plasma membranes, and infiltrated residual nodules. Satellite cells and IBA1-reactive monocytes displayed upregulated ferritin biosynthesis, which was most likely due to leakage of iron from dying neurons.

**Conclusions:**

We conclude that FA differentially affects the key cellular elements of DRG, and postulate that the disease causes loss of bidirectional trophic support between satellite cells and neurons.

## Introduction

Friedreich ataxia (FA) is an autosomal recessive disease with a prevalence in peoples of Western European (Caucasian) origin ranging from 1:43,000 to 1:140,000 [30]. The underlying mutation in the vast majority of cases is a homozygous guanine-adenine-adenine (GAA) trinucleotide repeat expansion in intron 1 of the *FXN* gene on chromosome 9q21.11. While the mutation causes a systemic deficiency of frataxin, a small mitochondrial protein, the clinical and neuropathological phenotypes are very diverse. In the central and peripheral nervous systems, FA affects motor cortex (Betz cells), dentate nucleus, spinal cord (dorsal nuclei in Clarke columns, dorsal columns, and dorsal spinocerebellar and corticospinal tracts), dorsal root ganglia (DRG), and sensory peripheral nerves [11, 12]. FA also causes hypertrophic cardiomyopathy, diabetes mellitus due to β-cell atrophy of the pancreas, kyphoscoliosis, and pes cavus (review in ref. [11]). Ataxia, dysarthria, dysmetria, dysphagia, weakness, flaccidity or spasticity, areflexia or hyperreflexia, peripheral neuropathy, hearing loss, visual impairment, and cognitive disability combine to form a severe neurological phenotype. The main cause of death, however, is cardiomyopathy. The reason for such diversity of lesions remains unknown, but lack of iron sulfur cluster (ISC) biosynthesis, incomplete ISC transfer to ISC-dependent proteins, deficient mitochondrial ATP production, and insufficient antioxidant defenses are under consideration for all affected tissues.

FA is often called a degenerative disease of neurons, including those of DRG, though frataxin deficiency also affects supporting cells. This report presents evidence for a primary disturbance of satellite cells and a role of inflammation in the destruction of DRG neurons in FA.

The principal methods in this work were immunohistochemistry and immunofluorescence with validated antibodies against structural and channel proteins of satellite cells and against inflammatory proteins. The current study benefitted from published data in experimental animals [4, 6, 7, 9, 17, 19, 22] and sought to establish relevant information in normal human DRG and the DRG in FA.

## Materials and methods

### Clinical data and autopsy specimens

The Institutional Review Board of the Veterans Affairs Medical Center in Albany, NY, USA, has approved the research described in this paper. DRG of 15 FA patients and 12 normal controls were available for routine staining of paraffin-embedded tissue sections, immunohistochemistry, and immunofluorescence. Tissues of FA patients were collected under a formal donation program supported by Friedreich’s Ataxia Research Alliance. Control DRG were obtained during autopsies conducted at Veterans Affairs Medical Center and Albany Medical College; and from National Disease Research Interchange, Philadelphia, PA, USA. Detailed clinical and genetic information was available for all patients (8 male, 7 female). Age of onset ranged from 2 to 18 years (mean ± standard deviation [S.D.]: 10 ± 5), and age of death from 10 to 69 years (mean ± S.D.: 36 ± 18). All patients had homozygous GAA repeat expansions, ranging from 249 to 1200 for GAA1 and 566–1200 for GAA2 (means ± S.D.: GAA1, 734 ± 251; GAA2, 955 ± 204). Autopsy delays were 2–96 h. The age range of the controls (9 male, 3 female) was 48–68 years (mean ± S.D.: 60 ± 6). Autopsy delays in the control cases ranged from 1 to 48 h.

### Immunohistochemistry and immunofluorescence

Paraffin sections of 6 μm thickness were processed to visualize selected proteins by immunohistochemistry and immunofluorescence. The overall approach was to visualize proteins in or around satellite cells with antibodies that were successfully used in animal experiments [4, 6, 7, 9, 17, 19, 22] or on human DRG [13, 15]. Table 1 provides detailed information on antibodies, sources, catalogue numbers (Cat. No.), and antigen retrieval methods. Details of immunohistochemistry and double-label immunofluorescence of DRG were described in previous publications [13–16]. Briefly, for immunohistochemistry, paraffin sections were rehydrated and oxidized in hydrogen peroxide-containing methanol, processed through antigen retrieval (Table 1), blocked by 10 % normal horse serum in phosphate-buffered saline (PBS), and incubated overnight at 4 °C in antibodies (Table 1) diluted in PBS, also containing 1 % normal horse serum. The next step was incubation at room temperature for 2 h in biotinylated anti-mouse, rabbit, or goat IgG (Vector Laboratories, Burlingame, CA USA), depending on the nature of the primary antibody. After repeated washing steps, the sections were immersed in a dilute solution of horseradish peroxidase-labeled streptavidin for 1 h, followed by a chromogenic solution of diaminobenzidine-urea-hydrogen peroxide (Sigma-Aldrich, St. Louis, MO, USA). Slides were dehydrated and cover-slipped by standard techniques, viewed in a Zeiss Axiophot microscope, and photographed. For immunofluorescence, sections were rehydrated in the same manner though oxidation by hydrogen peroxide in methanol was omitted. Antigen retrieval followed the plan outlined in Table 1 for each protein-of-interest. The suppressor protein was 10 % donkey serum in PBS (vol/vol). After incubation with primary antibodies, the sections were exposed to Alexa Fluor 488-labeled donkey anti-mouse IgG (Jackson ImmunoResearch Laboratories, West Grove, PA, USA) or Cy3-labeled donkey anti-rabbit or goat IgG (Jackson ImmunoResearch Laboratories). Sections were covered by a 50 % solution of glycerol in PBS (vol/vol). For selected pairs of Alexa Fluor 488- or Cy3-labeled antigens, 4,6′-diamidino-2-phenylindole (DAPI, 1.3 μmol/l) was added to the glycerol-PBS mixture to reveal nuclei. Sections were viewed and processed in a Zeiss LSM 510 Meta-NLO laser scanning confocal microscope. Exciting wavelengths for Alexa Fluor 488 and Cy3 were 488 nm and 543 nm, respectively. Band pass filters were 500–530 nm for Alexa Fluor 488 and 565–615 nm for Cy3. DAPI fluorescence was generated by two-photon technology at an exciting wavelength of 740 nm, emitted by a titanium-sapphire laser, and recorded through a band pass filter of 435–485 nm.

**Table 1:** Antigenic proteins, antibodies, and antigen retrieval methods

Antigenic protein	Antibody supplier	Catalogue number	Host	Clonality (isotype)	Protein concentration (μg/ml) or dilution for IHC or IF	Antigen retrieval method
ATP5B	Santa Cruz	sc-33618	mouse	monoclonal (IgG)	0.8 (IHC and IF)	tris (IHC), DIVA (IF)
CD68	Santa Cruz	sc-20060	mouse	monoclonal (IgG)	4 (IHC)	citrate
Connexin 43	Abcam	ab113470	rabbit	polyclonal (IgG)	0.75 (IHC and IF)	citrate
Cytosolic ferritin	GenWay	GWB-B74CB5	goat	polyclonal (IgG)	4 (IF)	chelation and DIVA
EAAT1/GLAST1	Santa Cruz	sc-77557	goat	polyclonal (IgG)	0.5 (IHC)	citrate
Frataxin	Abcam	ab110328	mouse	monoclonal (IgG)	10 (IHC)	tris
Glutamine synthetase	Santa Cruz	sc-74430	mouse	monoclonal (IgG)	1 (IHC)	citrate
IBA1/AIF1	Sigma	HPA049234	rabbit	polyclonal (IgG)	1 (IHC)	citrate (IHC), DIVA (IF)
Kir4.1	Alomone	APC-035	rabbit	polyclonal (IgG)	4 (IHC)	DIVA
Laminin	Sigma	L8271	mouse	monoclonal (IgG)	1:1000 (IHC)	proteinase K
mGluR2/3	Novus	NBPI-00924	rabbit	polyclonal ((IgG)	1 (IHC)	DIVA
S100	Santa Cruz	sc-53438	mouse	monoclonal (IgG)	0.4 (IHC), 0.8 (IF)	citrate
S100α	Santa Cruz	Sc-7849-R	rabbit	polyclonal (IgG)	0.4 (IHC)	citrate

*Abbreviations and notes*: *Abcam* Cambridge, MA, USA, *AIF1* allograft inflammatory factor 1 (synonym for IBA1), *Alomone* Alomone Labs, Jerusalem, Israel, *ATP5B* ATP synthase subunit beta, mitochondrial, *Chelation* preincubation of slides in 30 mM each of 2,2′-dipyridyl and sodium hydrosulfite in 0.1 M acetic acid-sodium acetate buffer, pH 4.8, *Citrate* 0.01 M citric acid/sodium citrate, pH 6, 10 min at 95 °C, 10 min cool-down period to room temperature, *DIVA* a proprietary antigen retrieval solution (Biocare Medical, Concord, CA, USA), diluted 1:10, 30 min at 95 °C, *EAAT1* excitatory amino acid transporter 1; synonym: GLAST1, glutamate aspartate transporter 1, *GenWay* GenWay Biotech, San Diego, CA, USA, *IF* immunofluorescence, *IHC* immunohistochemistry, *Kir4.1* ATP-sensitive inward rectifier potassium channel 10; gene product of KCNJ10, *Laminin* ascites fluid, *mGluR2/3* metabotropic glutamate receptors 2 and 3. The supplier reports that the polyclonal antibody raised against an mGluR2-specific peptide also reacts with mGluR3. mGluR2 and mGluR3 show 68 % sequence homology, *Novus* Novus Biologicals, Littleton, CO, *Proteinase K* 0.1 mg/ml, 0.1 M NaCl, 20 mM tris, pH 8, 30 min at 37 °C, *Santa Cruz* Santa Cruz Biotechnology, Santa Cruz, CA, USA, *Sigma* Sigma-Aldrich, St. Louis, MO, USA, *Tris* 0.1 M tris buffer, pH 9.6, 5 % urea (w/vol), 20 min at 95 °C

### Protein validation

We used several methods to validate the identity of proteins in tissue sections. Four proteins listed in Table 1 (ATP5B, connexin 43, EAAT1, and IBA1) were validated by tandem mass spectrometry including multiple reaction monitoring-initiated detection and sequencing (MIDAS), as described by Unwin et al. [28]. For this purpose, proteins in samples of frozen DRG (35–65 mg) were extracted by ultrasonication in a buffer found suitable for the assay of tissue frataxin [5, 16]. After centrifugation at 14,000 × g for 45 min, the supernatant was collected, and an aliquot was used for sodium dodecylsulfate polyacrylamide gel electrophoresis (SDS-PAGE) and Western blotting by established procedures. The apparent molecular weights of the detected protein bands were calculated by reference to a series of prestained standard proteins and compared with information on mass in UniProt [27]. The protein bands on matching Coomassie Blue-stained gels were excised for MIDAS. One or more signature peptides were identified for each of the four aforementioned proteins by in-gel trypsin digestion, alkylation, and MIDAS at the Center for Functional Genomics at State University of New York at Albany, Rensselaer, NY, USA. The authenticity of staining with antibodies to frataxin, laminin, glutamine synthetase, mGluR2/3, Kir4.1, CD68, and cytosolic ferritin was supported by absorption of the antibodies with the corresponding purified antigenic protein, recombinant protein, or antigenic peptide (frataxin: recombinant human protein, courtesy of Dr. Grazia Isaya, Mayo Clinic, Rochester, MN, USA; laminin: purified laminin, AbD Serotec, Raleigh, NC, USA, Cat. No. 5620–0604; glutamine synthetase: full length recombinant protein, Abcam, Cambridge, MA, USA, Cat. No. ab98145; mGluR2/3: peptide GREVVDSTTSSL, US Biological, Salem, MA, USA, Cat. No. M3884-76C; Kir4.1: antigenic peptide CKLEESLREQAEKEGSALSVR, Alomone Labs, Jerusalem, Israel, Cat. No. APC-035; CD68: recombinant protein, Abcam, Cat. No. ab38260; cytosolic ferritin: purified human liver ferritin, EMD-Millipore, Billerica, MA, USA, Cat. No. 341482). To further characterize S100 reaction products, we used a monovalent polyclonal anti-S100α antibody (Santa Cruz Biotechnology, Santa Cruz, CA, USA, Cat. No. SC-7849-R) and absorption of the monoclonal anti-S100 antibody by an excess of recombinant S100α (Abnova, Taipei, Taiwan, Cat. No. H00006271-P01) or full-length S100β-peptide (Abcam, Cat. No. ab30380).

## Results

### Structural proteins of satellite cells and gap junction protein connexin 43

**Fig. 1:**
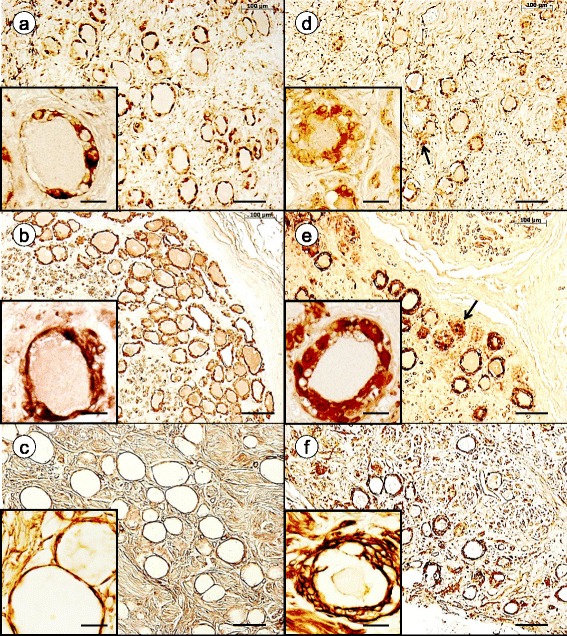
Immunohistochemistry of glutamine synthetase, S100, and laminin in DRG of normal controls and FA. **a**-**c** normal controls; **d**-**f** FA; **a** and **d** glutamine synthetase; **b** and **e** S100; **c** and **f** laminin. In normal DRG, reaction product of glutamine synthetase (**a**) and S100 (**b**) reveal well organized layers of adjacent and overlapping satellite cells around each neuron. Normal nerve cells are surrounded by delicate layers of laminin (**c**). In FA, the remaining neurons are smaller, and satellite cell layers are thicker (**d** and **e**, insets) and disrupted (**d**, inset). Laminin reaction product shows multiple layers surrounding neurons, which is consistent with proliferation of satellite cell processes (**f**, inset). The arrows in (**d**) and (**e**) point to residual nodules that are reactive with antibodies to glutamine synthetase (**d**) and S100 (**e**), respectively. Bars: (**a**-**f**), 100 μm; insets, 20 μm

Figure 1 illustrates satellite cell immunoreactivity with antibodies to glutamine synthetase, S100, and laminin. The general reduction in neuronal size in FA is apparent with all three stains, and satellite cell layers encircling neurons are disrupted (Fig. 1d, inset) or hypertrophic (Fig. 1e, inset). Glutamine synthetase and S100 immunoreactivity persist in residual nodules (Fig. 1d and e, respectively). Preabsorption of the monoclonal antibody against S100 by either recombinant S100α or full-length S100β peptide did not eliminate reaction product in satellite cells or residual nodules. Adding S100β-peptide to anti-S100, however, blocked immunoreactivity in Schwann cells of nerve bundles within DRG. We concluded that S100 reaction product in satellite cell cytoplasm represents a mixture of S100α and S100β as previously reported [1]. Polyclonal-anti-S100α generated reaction product of similar intensity in satellite cells but little if any in Schwann cells. Laminin reaction product in normal DRG shows a delicate rim outlining negative images of neurons (Fig. 1c) which changes to multiple stacked layers in FA (Fig. 1f).

**Fig. 2:**
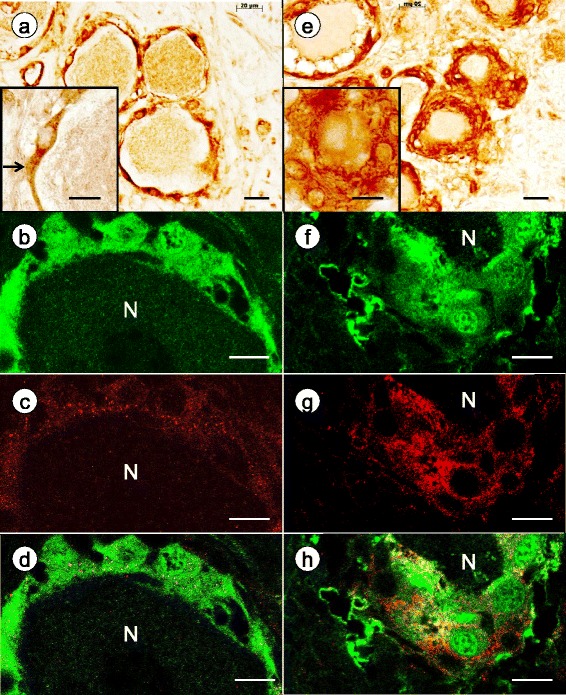
Immunohistochemistry and immunofluorescence of the gap junction protein connexin 43 and the satellite cell marker S100 in DRG of normal controls and FA. **a**-**d** normal control; **e**-**h** FA; **a** and **e** positive contrast immunohistochemistry of connexin 43; **b** and **f** Alexa 488 immunofluorescence of S100; **c** and **g** Cy3 fluorescence of connexin 43; **d** and **h**, merged images of **b** and **c**, and **f** and **g**, respectively. In the normal DRG, connexin 43 shows punctate and coalescing reaction product (**a**; and inset [arrow]). In FA, a great abundance of connexin 43 reaction product is present in multiple layers around small neurons (**e**). Hyperplastic satellite cells bridge gaps between neurons. The confocal images of a normal DRG show sparse gap junctions by their punctiform connexin 43 immunofluorescence (**c**). They are closely related to S100-positive satellite cells (**d**). In FA, the number of connexin 43-reactive puncta is greatly increased (**g** and **h**). Bars: (**a**) and (**e**), 20 μm; insets, 10 μm; (**b**-**d**) and (**f**-**h**), 10 μm. N, neuron

Figure 2 shows positive contrast immunohistochemistry of connexin 43 and double-label immunofluorescence of connexin 43 and S100 in DRG. Immunohistochemistry of a normal DRG (Fig. 2a) reveals punctate and coalescing reaction product in satellite cells. In FA, hypertrophic and hyperplastic satellite cells display increased expression of connexin 43 in multiple layers and become confluent with satellite cells surrounding other neurons (Fig. 2e). The double-label image of a normal DRG (Fig. 2d) reveals sparse connexin 43-positive gap junctions within a single-cell layer of S100-positive satellite cells. In FA, disorganized satellite cells display more abundant red connexin 43 fluorescence (Fig. 2g and h).

### Channel proteins of satellite cells

**Fig. 3:**
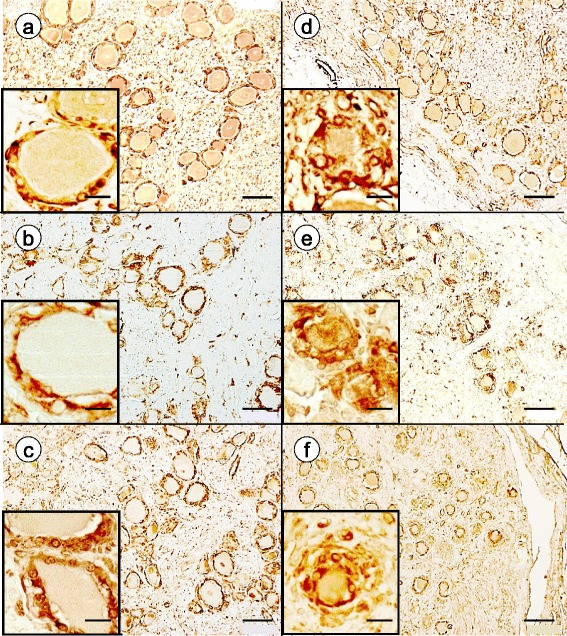
Immunohistochemistry of mGluR2/3, EAAT1, and Kir4.1 in DRG of normal controls and FA. **a**-**c** normal controls; **d**-**f** FA; **a** and **d** mGluR2/3; **b** and **e** EAAT1; **c** and **f** Kir4.1. In the normal controls, reaction product displays a single-cell satellite layer around each neuron ([**a**-**c**], insets). In FA, neurons are smaller and surrounded by a disorganized layer of multiple immunoreactive satellite cells. Bars: (**a**-**f**), 100 μm; insets, 20 μm

Immunohistochemistry of metabotropic glutamate receptors 2 and 3 (mGluR2/3), excitatory amino acid transporter 1 (EAAT1), and ATP-sensitive inward rectifying potassium channel 10 (Kir4.1) was used to gain insight into glutamate and potassium flux in satellite cells. Antibodies to these proteins generate prominent reaction product in normal satellite cells (Fig. 3a-c), and proliferating satellite cells and residual nodules in FA continue to express these proteins (Fig. 3d-f). The distribution of reaction product resembles that of glutamine synthetase and S100 (Fig. 1).

### Inflammatory cells and ferritin expression

**Fig. 4:**
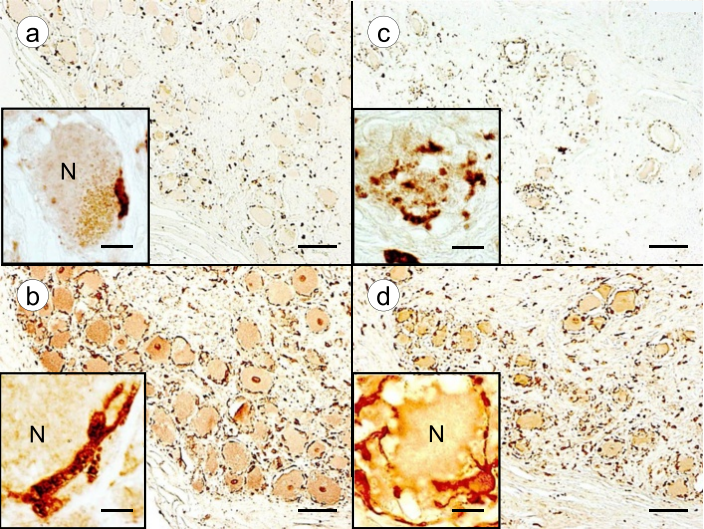
Immunohistochemistry of inflammatory cells in DRG of normal controls and FA. **a**-**b** normal controls; **c**-**d** FA; **a** and **c** CD68; **b** and **d** IBA1. **a** and **b** show the relative abundance of CD68- and IBA1-immunoreactive cells in normal DRG. Monocytes abut normal neurons (N) ([**a** and **b**], insets) but do not penetrate into the neuronal cytoplasm. In FA, a neuron shows invasion by CD68-positive monocytes resembling neuronophagia (inset) (**c**). Multiple IBA1-positive monocytes lie immediately adjacent to a degenerating nerve cell (N) (**d**). One ameboid monocyte appears to project thin processes along and through the neuronal plasma membrane (**d**, inset). Bars: (**a**-**d**), 100 μm; (**a**) and (**c**), insets, 20 μm; (**b**) and (**d**), insets, 10 μm

Normal DRG contain numerous CD68- and IBA1-reactive mononuclear cells (Fig. 4a and b, respectively). Some of these monocytes are located in close apposition to DRG neurons (Fig. 4a and b, insets). Of note, IBA1-reactive cells are more abundant than CD68-positive monocytes in normal and FA-affected DRG. CD68- and IBA1-immunoreactive cells participate in the direct invasion of neurons (Fig. 4c and d, insets). The image in Fig. 4c (inset) strongly resembles neuronophagia in the central nervous system.

**Fig. 5:**
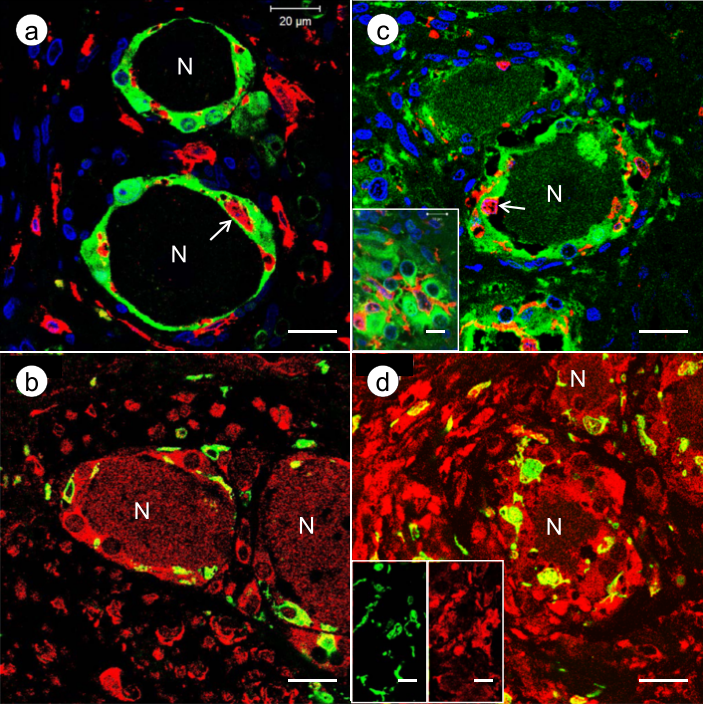
Double-label immunofluorescence of IBA1 and S100; and IBA1 and cytosolic ferritin in DRG of normal controls and FA. **a**-**b** normal controls; **c**-**d** FA. **a** and **c** double-label immunofluorescence of S100 (Alexa Fluor 488 green) and IBA1 (Cy3 red); DAPI (blue); **b** and **d** double-label immunofluorescence of IBA1 (Alexa Fluor 488 green) and cytosolic ferritin (Cy3 red). In the normal DRG, IBA1-reactive monocytes may gain access to the S100-reactive satellite cell sheath around neurons (N) but remain separated from the neuronal plasma membrane by a thin layer of satellite cell cytoplasm (**a**, arrow). IBA1 and S100 show no colocalization. In FA, the outline of the S100-positive satellite cell layer appears irregular and disrupted. An IBA1-reactive monocyte (arrow) abuts or penetrates the neuronal plasma membrane (**c**). The inset in (**c**) shows infiltration of a residual nodule by IBA1-positive monocytes. DAPI fluorescence in (**c**) confirms increased cellularity in FA when compared to the normal DRG (**a**). A normal DRG shows ferritin immunofluorescence in satellite cells and neurons (N) (**b**). The mixed yellow and green color suggests that ferritin biosynthesis also occurs in IBA1-positive monocytes. In FA, a multilayered rim of satellite cells around a shrunken neuron (N) is intensely fluorescent for cytosolic ferritin (Cy3 red) (**d**). Ameboid monocytes express both ferritin and IBA1. The inset in (**d**) shows matching single-color images in further support of the colocalization of IBA1 (Alexa Fluor 488 green) and ferritin (Cy3 red). Bars (all): 20 μm

Figure 5a and c display the localization of IBA1-positive monocytes in the context of S100-positive satellite cells of DRG. Invariably, IBA1-immunofluorescence is distinct from S100 localization in satellite cells. While IBA1-reactive cells may enter the layer of satellite cells surrounding a normal neuron, a thin sliver of S100-positive cytoplasm separates the monocyte from the neuronal plasma membrane (Fig. 5a). In FA, IBA1-positive monocytes pass through the disorganized satellite cell layer, encroach upon or penetrate the neuronal plasma membrane (Fig. 5c), and infiltrate residual nodules (Fig. 5c, inset). DAPI fluorescence discloses the increased cellularity of DRG in FA (Fig. 5c).

Figure 5b and d confirm the localization of cytosolic ferritin in the cytoplasm of neurons and satellite cells in DRG. Though the most intense red ferritin fluorescence arises from satellite cells, the yellow color on merged images in Fig. 5b and d suggests that IBA1-positive monocytes also contain this protein. Figure 5d of a DRG from an FA patient with a rapidly progressive course shows the great abundance of ferritin-positive satellite cells. The insets confirm the colocalization of IBA1 and cytosolic ferritin.

### Mitochondrial proteins

**Fig. 6:**
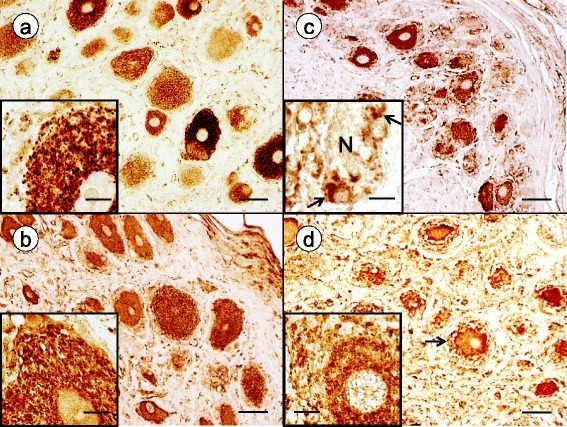
Immunohistochemistry of the mitochondrial proteins frataxin and ATP5B in DRG of normal controls and FA. **a**-**b** normal controls; **c**-**d** FA. **a** and **c** frataxin; **b** and **d** ATP5B. In a normal control DRG, frataxin reaction product is very prominent only in the neuronal cytoplasm (inset) whereas satellite and other cells in the space between neurons are almost devoid of immunoreactivity (**a**). In FA, satellite cells express abundant frataxin (**c**). The inset in (**c**) shows a neuron (N) that is devoid of frataxin while surrounding satellite cells are immunoreactive (inset, arrows). Similar to frataxin, ATP5B reaction product in mitochondria is prominent in neuronal cytoplasm of normal DRG (**b**, and inset). In FA, ATP5B reaction product continues to be present in the remaining small neurons but is more prominent in satellite cells (**d**, arrow, and inset). Bars (**a**-**d**), 50 μm; insets, 10 μm

Figure 6a and c show the distribution of frataxin reaction product in a normal DRG (Fig. 6a) and a DRG in FA (Fig. 6c). Expression of frataxin predominates in neurons of a normal DRG, and the space between nerve cells shows relatively little reaction product. In FA, many small surviving neurons remain frataxin-reactive, but expression of the protein becomes more prominent in proliferating satellite cells (Fig. 6c). A dead or dying neuron without frataxin reaction product may be surrounded by satellite cells that express this protein (Fig. 6c, inset). Immunohistochemistry of mitochondrial ATP5B (Fig. 6b and d) mirrors the distribution of frataxin (Fig. 6a and c). Normal neurons are rich in ATP5B reaction product whereas the surrounding satellite cell layer shows very limited expression (Fig. 6b, and inset). In FA, satellite cells contain many more ATP5B-positive mitochondria than normal (Fig. 6d, and inset).

## Discussion

### Limitations

Optimal preservation of tissue antigens depends on prompt fixation after death, which was difficult to achieve with autopsy delays beyond a few hours. It was evident that in some DRG samples, autolysis introduced a bias in the interpretation of immunohistochemistry of the more perishable antigenic determinants, and antigen retrieval methods (Table 1) did not always assure detection. Also, autolysis precluded ultrastructural study of satellite cells in FA.

The presence of reaction product does not necessarily provide information on satellite cell function or dysfunction. The emergence of tissue-based proteomics with multiple validated antibodies [7, 25], however, supports the conclusion that abnormal satellite cell structure correlates with expression changes in FA-related proteins.

### Satellite cell proliferation in FA

Invariably, FA causes hypercellularity of DRG, and immunohistochemical stains illustrated in Figs. 1, 2 and 3 confirm hypertrophy and hyperplasia of satellite cells. Residual nodules, also called nodules of Nageotte, retain immunoreactivity with antibodies to glutamine synthetase (Fig. 1d) and S100 (Fig. 1e) though their constituent cells have lost contact with DRG neurons. The differential expression of S100 in satellite cells (S100α and S100β) and nearby Schwann cells (S100β) is unexplained, but derivation of satellite cells from boundary cap cells may account for the dissimilar distribution of the S100 isoproteins [24]. Laminin reaction product displayed in Fig. 1f indicates that frataxin deficiency in FA causes the formation of redundant satellite cell processes in addition to an obvious numerical increase of these cells. Stains of glial fibrillary acidic protein and vimentin (not illustrated) resemble those of glutamine synthetase and S100. In none of the available DRG of 15 patients with FA did hematoxylin and eosin stains reveal mitoses, and immunohistochemistry with a monoclonal antibody to the KI-67 antigen was negative. Mitoses are present in developing DRG [20], but authors of systematic immunohistochemical studies of DRG in animals that underwent experimental manipulations did not comment on mitotic figures in proliferating satellite cells [4, 6]. Neuronophagic invasion (Fig. 4c and d) suggests an active process of nerve cell destruction that may be independent of slower satellite cell proliferation.

The greater abundance of connexin 43-reactive gap junctions in FA (Fig. 2e and g) correlates well with the increased number of satellite cell processes (Fig. 1f). Normal DRG neurons and their satellite cell layers may be viewed as units. When satellite cells proliferate, their processes contact cells surrounding neighboring neurons and form new gap junctions [8]. This sequence of events is apparent in FA (Fig. 2e). The biochemical stimulus to generate more gap junctions is not specific for FA, as a numerical increase of gap junctions occurs adult mice undergoing sciatic nerve transection [8].

### Glutamate and potassium flux in satellite cells of FA

Glutamate in DRG, glutamate recycling, and glutamate transport have received attention in pain processing by DRG in experimental animals [3, 17, 19] and in mixed neuronal/glial cell cultures [17]. The histochemical visualization of vesicular glutamate transporters in the neurons of DRG [3] implies biosynthesis and vesicular packaging of the neurohumoral transmitter in the perikarya of DRG neurons, though glutamatergic synaptic contacts occur at a distance in the spinal cord gray matter. Visualization of glutamine synthetase and EAAT1 in normal DRG (Figs. 1a and 3b, respectively) supports the hypothesis that neuronal glutamate recycling in DRG takes place in a transmembrane exchange between nerve and satellite cells [17]. A similar claim may be made for potassium flux control by Kir4.1 (Fig. 3c). The expression of mGluR2/3 in satellite cells (Fig. 3a), however, is more difficult to reconcile with neurohumoral transmission. Carlton and Hargett [4] suggested “modulation” of glutamatergic activity in DRG by mGluR. The persistence of glutamine synthetase, EAAT1, Kir4.1, and mGluR2/3 immunoreactivity in proliferating satellite cells provides only limited insight beyond a general disturbance of glutamate and potassium homeostasis in FA. In theory, glutamate dysmetabolism in DRG should cause more pain, which is not consistent with the moderate neuropathic symptoms in FA.

### Inflammatory infiltration of DRG in FA and ferritin expression in satellite cells

In his comprehensive review of sensory ganglia in animals, Pannese ([20]; page 61) commented only briefly on the presence of macrophages in normal DRG. Later studies utilizing immunohistochemistry of major histocompatibility complex II (MHCII) and ED-1 (a monoclonal antibody to CD68) confirmed the invariable presence of resident monocytes in DRG of normal animals [6, 9]. In the current study, CD68 (Fig. 4a) and IBA1 (Fig. 4b) reaction products indicate that resident monocytes are present and quite numerous in normal human DRG. The dissimilar expression of CD68 and IBA1 is unexplained. The discrepancy may be due to technical factors or the result of differential clonal expression. Dubový et al. [6] reported that in normal rat DRG, macrophages did not establish intimate contact with neurons. Sciatic nerve transection changed this relationship, and macrophages became neuronal “satellite cells”. It is unlikely, however, that cells expressing CD11, CD14, CD45, CD68, and MHCII in human trigeminal ganglia are actually neural crest-derived satellite cells [29]. The current study does not support expression of monocyte markers by satellite cells. While monocytes gain access to the satellite cell layer surrounding normal human DRG and mingle with satellite cells, S100 and IBA1 reaction products do not co-localize (Fig. 5a). Monocytes may lie very close to the neuronal plasma membrane in normal DRG, but high-resolution confocal immunofluorescence shows that they remain separated from the nerve cell (Fig. 5a). In FA, mononuclear cells appear to penetrate the neuronal cytoplasm in FA (Figs. 4d and 5c) though electron microscopy would be required for definitive proof of transmembrane migration. Monocytes also participate in the formation of residual nodules (Fig. 5c, inset). Cells with either CD68- or IBA1-expression participate in the destruction of DRG neurons (Fig. 4c and d, respectively). We did not observe polymorphonuclear leukocytes, CD3-, or CD20-positive lymphocytes in the inflammatory infiltrate of DRG in FA.

In FA, upregulation of ferritin biosynthesis in proliferating satellite cells is the likely response to Fe leakage from DRG neurons (Fig. 5d and ref [15]). It is remarkable that the main mechanism of ferritin biosynthesis is located in satellite cells rather than monocytes. These leukocytes and their derivative macrophages are fully capable of translating ferritin messenger ribonucleic acids in response to local Fe excess, and IBA1-reactive monocytes among proliferating satellite cells in FA are also ferritin-reactive (Fig. 5d, insets). Based on quantitative *in-situ* X-ray fluorescence, total DRG Fe in FA does not change, and retention of the metal is due to Fe uptake by satellite cells [15]. A similar process was also proposed for the transfer of Zn from DRG neurons to satellite cells, triggering the biosynthesis of metallothioneins 1 and 2 [15]. The evidence for a primary role of the two transition metals, Fe and Zn, in oxidative damage to DRG in FA remains inconclusive.

### Mitochondria in the satellite cells of DRG in FA

FA is a recognized mitochondrial disease, and the greater number of mitochondria in satellite cells requires an explanation beyond metabolic activation. It is plausible that the mitochondrial abundance is a compensatory, and likely maladaptive, response to oxidative stress, energy deficiency, or both (review in ref. [26]). Mitochondria are a source of reactive oxygen species, and an inappropriate number of these organelles in satellite cells may actually cause further damage to the cell. One conclusion of the work presented here is that frataxin deficiency does not impair mitochondrial biogenesis, but electron microscopy will be necessary to determine whether the ultrastructure of the “new” mitochondria in DRG of FA is entirely normal.

### Pathogenesis of the DRG lesion in FA

The literature contains few reports of normal or diseased human DRG that include immunohistochemistry and immunofluorescence. The rationale for our continuing work is to identify satellite cell proteins that undergo distinct changes in expression due to FA. The ultimate goal is to develop a more comprehensive tissue-based proteomic analysis [25] to measure the downstream effects of frataxin deficiency. Animal models of FA show vacuolation of DRG neurons [2, 23] but lack proliferation of satellite cells and residual nodules. From the published light-microscopic images of DRG in mouse models [2, 23], it cannot be inferred whether some of the cells surrounding vacuolated neurons are inflammatory. Pannese et al. [21] established that in DRG of cats and rabbits the volume of satellite cells is linearly related to volume and surface area of neurons. Rats and guinea pigs were not suitable for a comparable analysis because they did not meet the requirement of an approximately spherical shape of DRG neurons. For this reason, murine models of FA may not be suitable for a comparable study.

Visualization of satellite cell cytoplasm by multiple stains (Figs. 1, 2 and 3) leaves little doubt that the ratio of satellite cell volume to neuronal surface area in DRG of human FA is greatly increased. Hypertrophy of satellite cells (Fig. 1e), abundance of laminin (Fig. 1f), and upregulated mitochondriogenesis (Fig. 6c-d) militate against the interpretation that DRG hypercellularity is a passive phenomenon and simply due to collapse of ganglion cells. The functional consequences of excess satellite cell cytoplasm are unknown. A long-standing hypothesis states that satellite cells provide trophic support to DRG neurons [20, 21]. Nerve cells may also signal satellite cells, making trophic support bidirectional. It cannot be assumed that frataxin deficiency primarily impairs neurons. It is equally possible that satellite cell function fails and that atrophy and inflammatory invasion of neurons are secondary events.

A critical question in the pathogenesis of the DRG lesion in FA is: When does the disease process in DRG begin? The answer may arise from the following observations: The youngest patient in our group of 15, a boy, began to show symptoms at the age of 2 years and survived for only 8 more years because of aggressive FA cardiomyopathy. His DRG revealed neuronal atrophy, satellite cell proliferation, and inflammatory infiltration that were comparable to cases of much later onset. A systematic comparison of neuronal sizes in DRG of FA patients with juvenile-onset (< 20 years) and long survival (> 20 years), and late-onset (> 20 years) and long survival (> 20 years), did not reveal significant differences [13]. It may be concluded that quantifiable atrophy of DRG neurons does not correlate significantly with disease onset. In-situ hybridization of mouse embryos to detect frataxin messenger ribonucleic acid shows strong early expression in DRG [10], suggesting high metabolic demand for this protein and early, perhaps prenatal, vulnerability to FA.

Frataxin deficiency causes severe changes in sensory peripheral nerves [18], and it is uncertain that FA neuropathy is solely the result of the lesion in DRG. In theory, sensory neuropathy in FA may be a primary effect of the disease, contributing to the damage in DRG.

### Therapeutic considerations

Systemic or targeted replacement of frataxin is an obvious goal of therapy in FA. Koeppen et al. [13] expressed the opinion that FA causes hypoplasia and superimposed atrophy of DRG. Since onset of ataxia in FA does not correlate with the degree of neuronal atrophy in DRG, restoration of frataxin can only be expected to preserve residual DRG function. In contrast to DRG, neuronal loss in the dentate nucleus is progressive and more closely related to disease onset [14, 16]. The blood–brain barrier may block the delivery of frataxin protein or viral carriers of the *FXN* gene to the DN, but cells of DRG and sensory nerves should be more accessible. It is unknown whether anti-inflammatory drugs would improve survival of DRG neurons and benefit patients with FA neuropathy and sensory ataxia.

## Conclusions

Systematic immunohistochemistry and immunofluorescence of structural and channel proteins, and of proteins related to inflammation, permits a new interpretation of FA-related changes in DRG. The disease process in FA affects satellite cell independently and is not solely due to atrophy of DRG neurons. Satellite cells undergo hypertrophy and hyperplasia, maladaptive mitochondriogenesis, and enhanced ferritin expression. DRG are infiltrated by inflammatory cells that participate in the destruction of neurons similar to neuronophagia in the central nervous system. It is likely that frataxin deficiency causes loss of bidirectional trophic support of satellite cells and neurons.
